# Mutation of the Highly Conserved Ser-40 of the HIV-1 p6 Gag Protein to Phe Causes the Formation of a Hydrophobic Patch, Enhances Membrane Association, and Polyubiquitination of Gag

**DOI:** 10.3390/v6103738

**Published:** 2014-10-02

**Authors:** Friedrich Hahn, Christian Setz, Melanie Friedrich, Pia Rauch, Sara Marie Solbak, Nils Åge Frøystein, Petra Henklein, Jörg Votteler, Torgils Fossen, Ulrich Schubert

**Affiliations:** 1 Institute of Virology, Friedrich-Alexander-University, 91054 Erlangen, Germany; E-Mails: Friedrich.Hahn@viro.med.uni-erlangen.de (F.H.); Christian.Setz@viro.med.uni-erlangen.de (C.S.); Melanie.Friedrich@viro.med.uni-erlangen.de (M.F.); Pia.Rauch@viro.med.uni-erlangen.de (P.R.); Joerg.Votteler@biochem.utah.edu (J.V.); 2 Department of Chemistry and Centre for Pharmacy, University of Bergen, N-5007 Bergen, Norway; E-Mails: Sara.Solbak@kj.uib.no (S.M.S.); Nils.Froystein@kj.uib.no (N.Å.F.); Torgils.Fossen@kj.uib.no (T.F.); 3 Institute of Biochemistry, Charité Universitätsmedizin-Berlin, 10117 Berlin, Germany; E-Mail: Petra.Henklein@charite.de

**Keywords:** HIV-1, Gag p6, ubiquitination, virus budding, membrane association

## Abstract

The HIV-1 p6 Gag protein contains two late assembly (l-) domains that recruit proteins of the endosomal sorting complex required for transport (ESCRT) pathway to mediate membrane fission between the nascent virion and the cell membrane. It was recently demonstrated that mutation of the highly conserved Ser-40 to Phe (S40F) disturbs CA-SP1 processing, virus morphogenesis, and infectivity. It also causes the formation of filopodia-like structures, while virus release remains unaffected. Here, we show that the mutation S40F, but not the conservative mutation to Asp (S40D) or Asn (S40N), augments membrane association, K48-linked polyubiquitination, entry into the 26S proteasome, and, consequently, enhances MHC-I antigen presentation of Gag derived epitopes. Nuclear magnetic resonance (NMR) structure analyses revealed that the newly introduced Phe-40, together with Tyr-36, causes the formation of a hydrophobic patch at the C-terminal α-helix of p6, providing a molecular rationale for the enhanced membrane association of Gag observed* in vitro* and in HIV-1 expressing cells. The extended exposure of the S40F mutant to unidentified membrane-resident ubiquitin E3-ligases might trigger the polyubiquitination of Gag. The cumulative data support a previous model of a so far undefined property of p6, which, in addition to MA, acts as membrane targeting domain of Gag.

## 1. Introduction

The Gag polyprotein Pr55 of HIV-1 contains all structural components that are essential and sufficient for the formation of virus like particles (VLPs) [1,2]. Concurrent with virus release, Gag is processed by the virus encoded protease (PR) into its mature proteins matrix (MA), capsid (CA), nucleocapsid (NC), and the C-terminal p6 protein.

MA mediates targeting and binding of Gag to the plasma membrane and lines the inner shell of the mature virus particle [3,4]. CA forms the conical core encasing NC, which itself regulates the packaging and condensation of the viral genome into nascent virions [5,6]. The C-terminal p6 region of Gag is required to detach the nascent virion from the plasma membrane by the function of its two late assembly (l-) domains. The primary l-domain, the tetrapeptide motif PTAP, mediates the recruitment of TSG101 (tumor susceptibility gene 101) to the virus budding site [7,8,9,10]. The secondary l-domain consists of a YPX_3_L motif that regulates the binding of ALIX (ALG-2 interacting protein 1/X) to Gag [11,12,13,14]. Both proteins are components of the ESCRT (endosomal sorting complex required for transport) pathway that normally mediates topologically equivalent membrane fission events during membrane protein trafficking and cytokinesis [15,16,17,18,19]. In case of ESCRT-dependent sorting of membrane proteins into multivesicular bodies (MVBs), mono-ubiquitination and formation of K63-linked poly-ubiquitin chains serves as an important signal [20]. Consistently, monoubiquitinated species of Gag have been described [21,22] and are believed to play a certain, so far enigmatic, role in the budding process [7,23,24,25]. Mutation of the PTAP l-domain results, besides in a defective virus release, in an increased Gag ubiquitination which is believed to be a consequence of an accumulation of Gag at the plasma membrane [26].

In addition, p6 represents the predominant phosphoprotein in virus particles and is a substrate for several virus associated kinases, among them the atypical protein kinase C (aPKC) targeting the highly conserved serine residue 40, and Erk-2 (extracellular signal-regulated kinase 2) targeting Thr-23 [27,28,29]. The aPKC site Ser-40 is somehow important for CA-SP1 processing, morphogenesis of progeny virions and virus maturation [30,31], but the molecular mechanism how this conserved Ser-40 regulates those late processes in virus replication still remains enigmatic. However, and unlike l-domain mutants, the exchange of Ser-40 to Phe (S40F) causes no defect in virus release [30,31], but specifically induces Gag-containing membrane protrusions resembling filopodia [31]. Moreover, the ALIX-interacting protein syntenin [32], which is known to bind both K48- and K63-linked polyubiquitin [33,34], has been speculated to play a role in the process of S40F-mediated filopodia formation [31]. In addition, the S40F mutation interferes with the Nedd4 ubiquitin ligase mediated virus release [31]. Because it is also well established that phosphorylation can generally trigger ubiquitination [35], these cumulative results prompted us to investigate the interaction of S40F mutant Gag with the ubiquitin proteasome system (UPS).

Here we show that the S40F mutation specifically leads to the formation of a hydrophobic patch on the C-terminal α-helix of p6 which consequently enhances the membrane interaction of Gag. This augmented membrane association of the S40F mutant is accompanied, similar to the mutation of the PTAP l-domain, by increased K48-linked polyubiquitination of Gag and, thus, enhances its entry into the UPS. This phenomenon provides evidence for an l-domain independent induction of Gag ubiquitination that might be causally linked to the formation of a cryptic membrane interaction site within the C-terminal α-helix of p6. In addition, the cumulative data support a previously hypothesized model [36] whereby, in addition to MA, p6 inserts in plane into the lipid bilayer and thus regulates the membrane association of Gag.

## 2. Materials and Methods

### 2.1. Cell Culture and Transfection

HeLa cells were cultivated in DMEM supplemented with 10% (v/v) inactivated fetal calf serum (FCS), 2 mM l-glutamine, 100 U/mL penicillin, and 100 μg/mL streptomycin. HeLa-K^b^ cells were maintained in DMEM with additional 1 mg/mL geneticin. B3Z cells were cultured in RPMI 1640 with 10% (v/v) FCS, 2 mM l-glutamine, 100 U/mL penicillin, 100 g/mL streptomycin, 0.01% (v/v) sodium pyruvate, and 10 mM HEPES. All cell culture reagents and media were purchased from Gibco (Life Technologies, Carlsbad, CA, USA). 

Confluent monolayers of HeLa cells were transfected with Lipofectamine 2000 (Life Technologies, Carlsbad, CA, USA) using the manufacturers protocol. 24 h post transfection cells were lysed in RIPA buffer (150 mM NaCl, 50 mM Tris-HCl pH 8.0, 1% NP-40, 0.5% Na-deoxycholate, 0.1% SDS, 10 mM EDTA) containing protease inhibitor cocktail complete mini (Boehringer, Ingelheim am Rhein, Germany), 5 mM N-ethylmaleimide (NEM), and 1 mM phenylmethylsulfonylfluoride (PMSF) and protein samples were added to equal volumes of sample buffer and separated by SDS PAGE. Virus containing supernatants were centrifuged through 20% sucrose cushions (w/v) for 90 min at 20,000 × *g*. Pelleted VLPs were washed with 1 ml cold PBS and lysed in sample buffer.

### 2.2. Detection of Ubiquitinated Gag

HeLa cells were lysed with RIPA buffer containing protease inhibitor cocktail NEM, and PMSF. Lysates were adjusted to 1% SDS (w/v) and incubated at 95 °C for 10 min, subsequently diluted to 0.1% SDS and were then cleared by centrifugation at 20,000 × *g* for 10 min. Gag content was recovered by immunoprecipitation with antibodies derived from HIV-1 patient sera pre-bound to GammaBind Plus Sepharose (GE Healthcare, Little Chalfont, United Kingdom). For detection of ubiquitinated Gag proteins directly by Western blotting cells were lysed with RIPA with 20 μM lactacystin and 20 μM carbobenzoxyl-leucine-leucine-leucinal and soluble extracts were analyzed by Western blotting.

### 2.3. SDS PAGE and Western Blotting

Protein samples were separated by SDS-PAGE [37] and subsequently transferred onto PVDF membranes (GE Healthcare). Membranes were blocked with 3% bovine serum albumin and incubated with the appropriate primary antibody (Ab). HA-tagged ubiquitin was visualized using an HA-reactive monoclonal antibody (mAb) directly conjugated to horse radish peroxidase (HRP) (Roche, Basel, Switzerland). FLAG-ALIX was detected by an *anti-*FLAG mAb directly conjugated to HRP (Sigma, St. Louis, MO,USA). Gag was detected by a rabbit Ab recognizing p24 (Seramun, Heidesee, Germany). The *anti-*mouse and *anti-*rabbit secondary antibodies coupled to HRP were obtained from Dianova (Dianova, Hamburg, Germany). Protein bands were quantified using AIDA (Raytest, Straubenhardt. Germany). For the linkage specific staining of polyubiquitin the rabbit mAb Apu2 (Millipore, Darmstadt, Germany) was used for the detection of K48-linked polyubiquitin and the mouse mAb HWA4C4 (Enzo, Farmingdale, NY, USA) for K63-linked polyubiquitin. Recombinant polyubiquitin chains either linked via the internal lysine 48 or 63 (Boston Biochem, Cambridge, MA, USA) served as specificity control. The mouse anti-Transferrin receptor antibody was purchased from Life Technologies and the anti-β-actin antibody from Sigma.

### 2.4. Expression Plasmids

The plasmid for expression of hemagglutinin (HA)-tagged ubiquitin (HA-Ub) was kindly provided by H.-G. Kräusslich and is described elsewhere [38]. The FLAG-ALIX expression construct is described elsewhere [39]. The NL4-3 [40] derived expression constructs pNLenv1 [41] and p∆R [38] harboring the S40F and ∆PTAP mutations have been previously described [30], the S40F/∆PTAP double mutant has been established analogously. All point mutations were introduced by site-directed mutagenesis (QuikChange^®^ Lightning, Life Technologies) using a pair of complementary primers (only forward primers are annotated). Mutational inactivation of the viral protease (PR^−^) was carried out using the primer pair 5’-gga agc tct att agc tac agg agc aga tga-3’. Alternatively Ritonavir was added to a final concentration of 10 μM 4 h post transfection to inhibit Gag processing. The S40D mutation was introduced by using the primer pair 5’-act gta tcc ttt agc cga cct cag atc act ctt-3’, the S40N mutation using the primer pair 5’-act gta tcc ttt agc caa cct cag atc act ctt-3’, and the N-ctrl construct using the primer pair 5’-act gta tcc ttt agc cag cct cag atc act ctt-3’. The MA G2A mutation was introduced by using the primer pair 5’-aga agg aga gag atg gct gcg aga gcg tcg gta-3’. The codon optimized Gag expression plasmids containing the SL sequence with *wt* p6 sequence and exchange of the two PTAP motifs have been described elsewhere [42,43]. Introduction of the S40F mutation in the syngag background was achieved by site-directed mutagenesis using the primer pair 5’-ctg tac ccc ctg acc ttc ctg agg agc ctg ttc ggc-3’, the S40A mutation by using the primer pair 5’-ctg tac ccc ctg acc gcc ctg agg agc ctg ttc ggc-3’, and the Y36A mutation with the primer pair 5’-cga caa gga gct ggc ccc cct gac c-3’.

### 2.5. Flow Cytometry

For detection of H2-K^b^-bound SL-epitope, cells were stained with the allophycocyanin (APC)-conjugated 25D1.16 mAb (eBioscience, San Diego, CA, USA) diluted 1:100 in FACS buffer (5% [v/v] FCS, 0.02% [v/v] NaN_3_ in PBS). For detection of total H2-K^b^ molecules, cells were incubated with hybridoma cell culture supernatant containing the mAb B8-24-3 [44], followed by staining with secondary Alexa 647-conjugated *anti-*mouse Ab (Life Technologies). For intracellular Gag staining, cells were permeabilized using Cytofix/Cytoperm (BD Bioscience, San Jose, CA, USA). Gag was detected by staining with a FITC-conjugated *anti-*p24 Ab (KC57; Beckman Coulter, Brea, CA, USA) diluted 1:100 in Perm/Wash buffer (BD Bioscience). Flow cytometry was performed on a FACSCalibur using CellQuest software (BD Bioscience). Data were analyzed by using the FACS Express V3 software [45].

### 2.6. T-Cell Activation Assay

The SL-H2-K^b^-specific murine CD8^+^ hybridoma T cells B3Z express the *lac*Z reporter gene under the control of the NFAT enhancer [46]. HeLa-K^b^ cells transfected with syngag plasmids coding for Gag *wt*, Gag-SL *wt*, ∆PTAP-SL, S40F-SL, or S40F/∆PTAP-SL were detached and seeded in triplicates into 96 well plates, in concentrations ranging from 50,000 to 150 cells/well. After overnight coculture with 50,000 of B3Z cells in a total volume of 200 μL/well, cells were washed twice with PBS, lysed by addition of 100 μL of 0.15 mM chlorophenyl red β-galactopyranoside (CPRG), 0.5% NP-40 in PBS and the absorbance at 595 nm was determined in a plate reader (BioTek Instruments, Bad Friedrichs-hall, Germany).

### 2.7. Membrane Flotation

The protocol for membrane flotation was adapted from [26]. Briefly, HeLa cells were extensively washed and scraped in homogenization buffer (0.25 M sucrose, 1 mM EDTA, 20 mM Tris HCl pH 8.0) and lysed by sonication. The post-nuclear supernatant was adjusted to 40% OptiPrep and underlayed to 28% and 2.5% OptiPrep. Samples were centrifuged at 109,000 × g for 3 h. Five fractions were collected from the top of the gradient and subsequently analyzed by Western blotting.

### 2.8. Peptide Synthesis

Synthesis and purification of synthetic p6 fragments, p6(23-52)S40F, p6(23-52)S40N and p6(23-52)S40D were performed as described in detail elsewhere [47,48].

### 2.9. Preparation of Samples for NMR Spectroscopy

The peptides were dissolved without pH adjustment (pH ~ 3.0) to final concentrations of 1–2 mM in 1:1 mixtures of H_2_O and CF_3_CD_2_OH (50% aqueous TFE-D_2_). TFE-D_2_ (99.5%) was purchased from Sigma.

### 2.10. NMR Spectroscopy

Two-dimensional (2D) ^1^H correlation spectroscopy (COSY), total correlation spectroscopy (TOCSY) and NOESY NMR spectra of the C-terminal p6 peptides were recorded without spinning at 300 K on a Bruker Avance 600 MHz instrument equipped with an UltraShield^TM^ Plus magnet and a triple resonance cryo-probe head with gradient unit. Data acquisition, processing and spectral analysis were in all cases performed with standard Bruker software [49]. All spectra were internally referenced to the residual TFE-DH signal at 3.95 ppm. The applied mixing times for the TOCSY and NOESY experiments were in all instances 110 ms and 250 ms, respectively. The unambiguous amino acid spin systems and the sequential assignments were established using a standard procedure [50] combining homonuclear 2D ^1^H TOCSY and 2D ^1^H NOESY NMR spectral data. Individual spin systems were identified from 2D ^1^H TOCSY spectra, starting from the backbone amide protons and sequence-specific assignments were determined from cross-peaks in the 2D ^1^H NOESY spectra based on short observable distances between ^1^H_N_, ^1^H_α_ and ^1^H_β_ nuclei of amino acid residue *i* and ^1^H_N_ of residue *i* + 1. This procedure provided the unambiguous assignments of all the spin systems for each of the mutant C-terminal p6 peptides (Tables S1-S3) and allowed a direct comparison with the data for the *wt* peptide reported previously under the same solution conditions [48].

### 2.11. Structural Calculations

The crosspeaks observed in the 2D ^1^H NOESY spectrum of p6(23-52)S40F were assigned and integrated using Sparky NMR Analysis version 3.1.1.4 [51]. Distances were derived from NOE peak volumes in the ^1^H NOESY spectrum and structure calculations were performed by using CYANA 2.1 [52]. The NH_i_ - NH_i+1_, NH_i_ - NH_i+2_, αH_i_ - NH_i+2_, αH_i_ - NH_i+3_, αH_i_ - NH_i+4_ and αH_i_ - βH_i +3_ crosspeaks observed in the NOESY spectrum were used for structure calculation. 100 structures were calculated. The calculated structures were processed and aligned with PyMOL software [53]. The residues I31-D48, which are included in the C-terminal helix of p6, were chosen as fitting regions for alignment of the 20 best structures (Figure S1).

## 3. Results

### 3.1. Mutation of Ser-40 Elevates Gag Ubiquitination

Recently, we and others reported that specific mutation of the highly conserved serine 40 to phenylalanine (S40F) within p6 leads to impaired CA-SP1 processing resulting in an abnormal core morphology and thus in a reduced replication capacity, whereas virus release and binding of ALIX to p6 were not reduced [30,31]. Later, it was reported by others that this S40F mutation induces filopodia-like membrane protrusions harboring Gag [31]. However, the mechanism by which mutation of Ser-40 in p6 affects CA-SP1 processing, virus morphology, and filopodia formation remained completely unclear. It has been shown that Ser-40 is phosphorylated in the context of the Gag precursor Pr55 by aPKC and that about overall 5-10% of the mature p6 protein is phosphorylated in virus particles [27,28,54]. Since phosphorylation is known to trigger ubiquitination [35], we investigated whether Ser-40 affects this modification of Gag, which was previously shown to be dependent only on the integrity of the PTAP l-domain [26,38,55].

**Figure 1:**
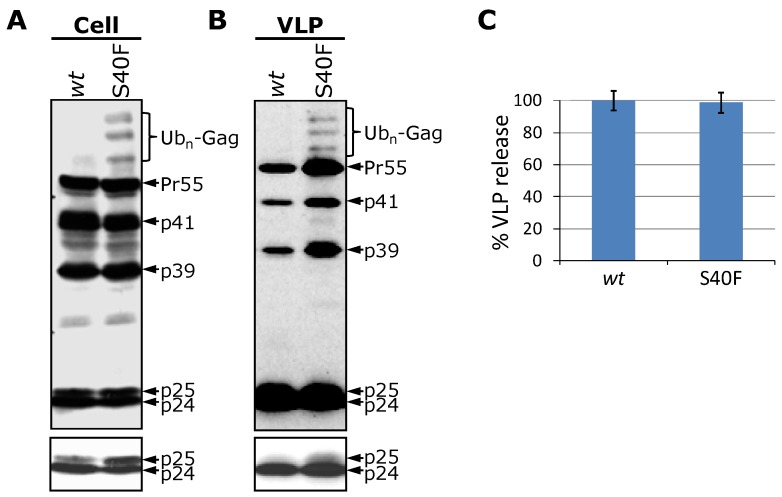
The S40F mutation in p6 augments Gag ubiquitination. HeLa cells were transfected with the *env*-deleted HIV-1 expression plasmid pNLenv1, as *wt* or S40F mutant, and lysed under conditions where the 26S proteasome and ubiquitin hydrolases were inhibited. Cell lysates (**A**) and VLP fractions (**B**) were analyzed by Western blotting using a CA-reactive antiserum. Shorter exposures of the Western blots revealing the delay in CA processing for the S40F mutant are depicted in the lower panels (**C**). The VLP release was quantified by calculating the amount of CA in the VLP fraction* versus* the amount of total Gag detected in cells and VLPs. Bars represent mean values of seven independent experiments ± SD. Values on the y-axis were adjusted that *wt* matches 100%.

Western blot analyses of HeLa cell lysates derived from cells transfected with the subgenomic HIV-1 expression construct pNLenv1 [41] directing the expression of *wt* and the S40F mutant reveal a ladder of bands migrating above Pr55 for the S40F mutant, which are reminiscent of ubiquitinated Gag species (Figure 1A). The same analyses of virus like particle (VLP) preparations displayed a very similar pattern of high molecular weight species of Gag for the S40F but not for the *wt* VLPs (Figure 1B). Quantification of the amount of CA in the VLP fraction* versus* the total amount of Gag confirmed the previously reported wild type efficiency for virus release of the S40F mutant (Figure 1C) [30,31]. Enhanced Gag ubiquitination in the context of normal virus release and intact l-domain functions is unprecedented, as increased Gag ubiquitination until now has only been described for l-domain mutants of HIV-1 [26,38,55]. The previously reported CA-SP1 processing defect of the S40F mutant [30,31] is visible upon shorter exposure of the Western blot membrane (Figure 1A,B, lower panels).

**Figure 2:**
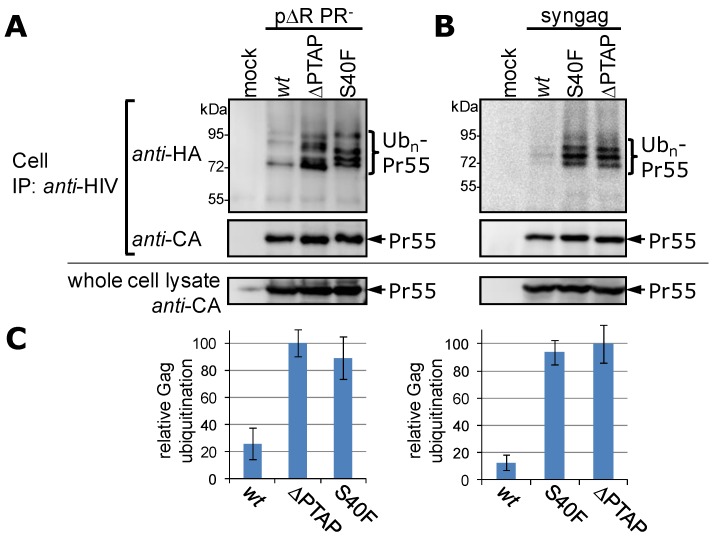
The S40F mediated ubiquitination of Gag occurs irrespective of Gag processing or other viral proteins. HeLa cells were co-transfected with HA-ubiquitin and either p∆R PR^−^ plasmids (**A**) or syngag plasmids encoding for either *wt* Gag, or the p6 mutant S40F or ∆PTAP, respectively (**B**). Gag was recovered by immunoprecipitation from denatured whole cell lysates with *anti*-HIV antibodies, and ubiquitinated Gag molecules were subsequently visualized by Western blot using antibodies specific for HA. The amount of Gag recovered by immune precipitation, as well as Gag recovered from whole cell lysates was detected with CA specific antibodies; (**C**) Evaluation of three or five independently performed experiments for the p∆R or the syngag system, respectively. Values were matched to the ubiquitin signal of the ∆PTAP mutant (100%) and represent the arithmetic mean ± SD.

To confirm that this ladder of higher molecular weight species indeed represents ubiquitinated Gag proteins, HeLa cells were co-transfected with Gag expression constructs and a plasmid encoding HA-tagged ubiquitin (HA-Ub). As control, we included the ∆PTAP l-domain mutant where the tetrapeptide motif PTAP is replaced by LIRL [10,56], a mutant which was previously shown to exhibit high levels of Gag ubiquitination in cells [26,38,55]. As Watanabe* et al.* observed the formation of filopodia-like structures for the S40F mutant especially in the context of unprocessed Gag, we employed a PR-deficient subgenomic HIV-1 expression construct that harbors an inactive RT and a primer binding site deletion (p∆R PR^−^ [38]). Additionally, by this approach we wanted to ensure comparable levels of Pr55 polyprotein as the substrate for ubiquitination, because the ∆PTAP mutation causes a Gag processing defect leading to accumulation of cell associated Pr55, a phenotype which is not observed for the S40F mutant, which shows *wt* Gag processing except for the CA-SP1 cleavage (Figure 1B). Gag was precipitated from whole cell lysates with antibodies derived from pooled HIV-1 patient sera. Ubiquitinated Gag was specifically detected by Western blotting using an HA-reactive antibody. Results shown in Figure 2A confirm that the ladder observed for the S40F mutant consists of ubiquitinated Gag species and that this modification occurs to a similar extent as for the ∆PTAP mutant.

It was suggested by Watanabe* et al.* that Gag alone is sufficient for the formation of S40F-induced filopodia [31]. To investigate whether the S40F mediated Gag ubiquitination occurs independently of other viral proteins, we compared the S40F and ∆PTAP mutants in context of a codon optimized synthetic *gag* gene expressed from a CMV promoter driven expression plasmid (p*syngag*) [42,57]. Introduction of the S40F mutation into the p*syngag* background enhances the Gag ubiquitination at a similar rate when compared to the situation when Gag was expressed in the context of the HIV-1 genome (Figure 2B). Again, the level of Gag ubiquitination for the S40F mutant was almost indistinguishable from that observed for the ∆PTAP mutant. As in this experimental setting the turnover of polyubiquitinated Gag by either the 26S proteasome or deubiquitinating enzymes is prevented, also the formation of ubiquitinated Gag species was observed in the *wt* situation, although at much lower level, approximately 10%–20% of the Gag-ubiquitin adducts detected for S40F and ∆PTAP as measured by densitometry (Figure 2C).

Therefore, the S40F mutant exhibits Gag ubiquitination independently of Gag processing or other HIV-1 proteins, and this ubiquitination occurs to an extent in cell and VLP fraction that is comparable to that of the ∆PTAP mutant.

### 3.2. The Non-Conservative Amino Acid Exchange S40F, but Not the Conservative Substitutions S40D or S40N, Induces Elevated Gag Ubiquitination

It has been shown that p6, as a substrate for several virus associated kinases, is the predominant phosphoprotein in virus particles [27]. However, it has only been demonstrated that the phosphorylation of Thr-23 by Erk-2 is important for budding and maturation of virus particles [29], whereas the aPKC mediated phosphorylation of Ser-40 facilitates Vpr incorporation [28].

**Figure 3:**
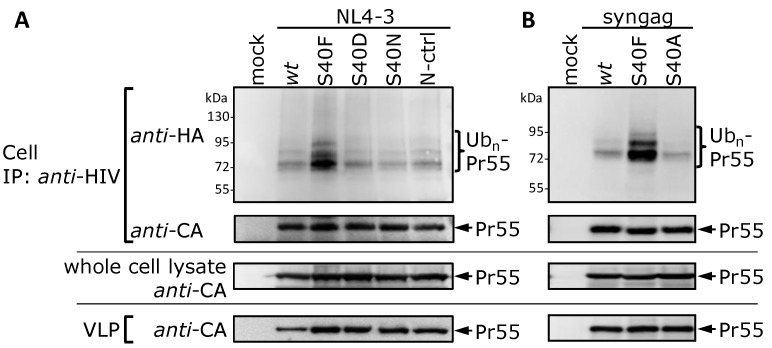
Influence of the S40N, S40D or S40A mutation on Gag ubiquitination. (**A**) HeLa cells were co-transfected with HA-tagged ubiquitin and pNL4-3 plasmids, as indicated. Gag processing was inhibited by the addition of the PR-inhibitor Ritonavir. The isolation and detection of ubiquitinated Gag species from cell fractions were carried out as described in Figure 2. The amount of Gag recovered by either immune precipitation or from whole cell lysates, and the VLP fraction was detected with CA specific antibodies; (**B**) HeLa cells were co-transfected with HA-tagged ubiquitin and the *syngag* expression plasmids directing the expression of *wt* Gag and mutants thereof. The isolation and detection of ubiquitinated Gag species from cell fractions were carried out as described in Figure 2. The amount of Gag recovered by immune precipitation, Gag recovered from whole cell lysate, and the VLP fraction was detected with CA specific antibodies.

Based on our observation (Figure 1 and Figure 2), it was intriguing to speculate that phosphorylation of Ser-40 by aPKC might trigger, or at least somehow regulate, the ubiquitination of Gag, yet the hydrophobic Phe cannot mimic at all a phosphoseryl residue. To more directly analyze a potential correlation between phosphorylation and ubiquitination, we established an S40D mutant in the context of the HIV-1 NL4-3 proviral clone [40]. The negative charge of the aspartic acid should function as phosphomimetic and thus simulate a constitutively phosphorylated serine in position 40. Additionally, we included an S40N mutant as a more conservative exchange for a phosphoseryl residue, which was also chosen by Radestock* et al.* in previous functional studies on p6 phosphorylation [54]. Since Ser-40 in p6 can only be exchanged to Phe without disturbing the amino acid sequence encoded by the overlapping *pol*-ORF [30], the p6 S40N mutation results in a Phe to Gln exchange in position 56 in the Pol-protein. As control, this mutation in Pol was established in the NL4-3 background while leaving the p6 amino acid sequence unchanged (N-ctrl). However, in case of the S40D mutant the resulting Phe to Arg mutation in position 56 in the Pol-protein could not be introduced in the context of the *wt* p6 sequence due to constraints of the codon usage and the overlapping ORFs. As particularly Ser-40 in p6 overlaps with the cleavage site between the transframe p6* protein and PR in the *pol*-ORF, we expected that the exchanges in the *pol*-ORF might interfere with the PR activity. To circumvent this potential problem, we applied the PR-inhibitor Ritonavir to yield comparable amounts of Pr55. However, analyses of the cell-associated Gag ubiquitination revealed that none of the conservative mutants, S40N or S40D, can increase Gag ubiquitination as it was observed for the S40F mutant (Figure 3A). In addition, also the N-ctrl mutant exhibits *wt* Gag ubiquitination (Figure 3A) indicating that the phenomenon is not related to changes in the overlapping *pol*-ORF. Our suspicion that this phenomenon is somehow specific for S40F, was further supported by recent finding from Watanabe* et al.*, demonstrating that the S40A mutant displays *wt* CA-SP1 processing [31]. In consistency, Western blot analyses revealed that the S40A mutation had, as for the S40N and S40D mutations, no obvious impact on ubiquitination of Gag (Figure 3B). Moreover, comparable amounts of Gag were detected intra- and extracellularly (cell and VLP fraction, Figure 3A,B, lower panels), revealing no decrease in virus release.

Thus, we conclude that the enhanced Gag ubiquitination of the S40F mutant, like the formation of the filopodia-like structures and the defective CA-SP1 processing, represents most probably a Phe specific phenomenon.

### 3.3. The S40F Mutation Increases the Amount of Membrane Associated Gag

First* in vitro* evidence that in HIV-1 expressing cells the introduction of the S40F mutation somehow increases the amount of Pr55 in membrane associated assembly compartments was reported previously [58]. Therefore an enhanced membrane localization of Gag could possibly account for its elevated levels of ubiquitination according to the model for ubiquitination of ∆PTAP mutants by membrane resident ubiquitin ligases [26].

**Figure 4:**
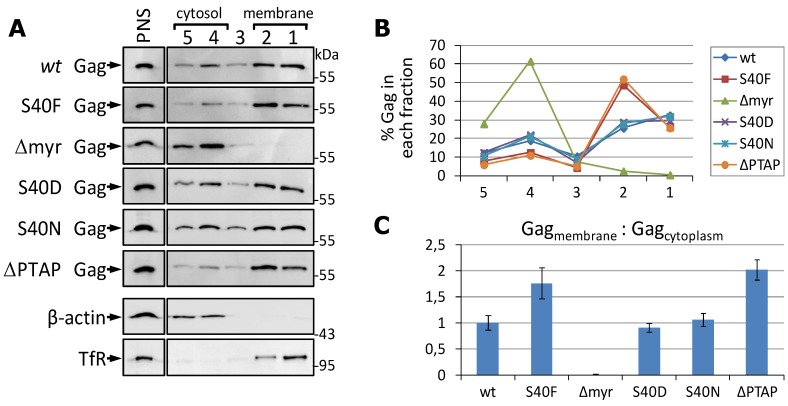
The S40F mutation increases selectively the membrane association of Pr55. (**A**) HeLa cells were transfected with p∆R PR^−^ HIV-1 constructs directing the expression of either *wt* Gag, the p6 S40F, S40N, S40D or ∆PTAP mutants or the MA G2A mutant. The cells were lysed by sonication and the post-nuclear supernatant (PNS) was subjected to membrane flotation. Fractions were collected and analyzed by Western blotting for Gag. The Western blot membrane was reprobed with antibodies specific for transferrin receptor (TfR) and β-actin as marker proteins for the membrane or cytosolic fractions, respectively. One representative example of TfR and β-actin blots is shown; (**B**) Band intensities of the Gag blots were densitometrically quantified and the distribution of Gag was determined for each mutant; (**C**) The ratio of Gag detected in the membrane fractions (fractions 1 and 2)* versus* the cytosolic fraction (fractions 4 and 5) was calculated. Values represent the mean of 4 independent experiments ± SD, and were adjusted that *wt* matches 1.

Thus, we investigated the impact of the S40F mutation on the intracellular distribution of Gag in PR-deficient context by membrane flotation, an established method that has been previously applied to address the membrane binding properties of Gag mutants [26,38,59]. The amount of Gag in each fraction was detected by Western blotting. As a control, we used the MA G2A mutation, which abrogates the myristoylation of Gag, and, thus, strongly decreases membrane binding [60], and the ∆PTAP mutant, which was shown previously to exhibit increased amounts of membrane bound Gag [26]. Detection of transferrin receptor and β-actin by Western blotting revealed a complete separation of both marker proteins and identified fractions 1 and 2 as the membrane-containing fractions and fractions 4 and 5 as cytosolic fraction (Figure 4A).

In consistency with previously reported surface plasmon resonance analyses [58], only the mutant S40F, but none of the conservative mutations S40D and S40N, increases the membrane associtation of Gag when compared to *wt* (Figure 4A). Quantification of the band intensities revealed that the S40F mutant exhibits a distribution of Gag within the gradient which is comparable to that of the ∆PTAP mutant (Figure 4B). Calculation of the ratio between membrane (fractions 1 and 2) and cytoplasmic (fraction 4) Gag from four independent experiments revealed a significant 1.75-fold increase of membrane associated Gag for the S40F mutant when compared to *wt* protein or the S40D and S40N mutants (Figure 4C).

Altogether, these data demonstrate that the S40F mutant enhances the membrane association of Gag at a rate comparable to a ∆PTAP mutant. Albeit different to the ∆PTAP, the S40F mutant exhibits *wt*-like virus release. Thus, CA-SP1 processing, virus maturation, membrane interaction, and Gag ubiquitination appear to be connected to each other, inasmuch as only the S40F, but none of the investigated conservative mutations, influence those processes.

### 3.4. Augmentation of the VLP Release by ALIX Does Not Affect Gag Ubiquitination

Next, we wanted to investigate the consequences of ALIX overexpression on Gag ubiquitination, which was demonstrated to restore virus release of PTAP-deficient Gag mutants while being inactive on release of the S40F mutant [12,13,30]. It was hypothesized previously that ubiquitination of Gag occurs primarily by plasma membrane resident ubiquitin ligases, particularly when Gag accumulates there in the case of ∆PTAP mutants [26,38,55]. Thus, in the reverse case, restoring virus release of a PTAP-deficient mutant by overexpressing ALIX should reduce Gag ubiquitination, a question that has not been addressed yet. Additionally, as an increased ALIX-binding has been implicated in the formation of the S40F-induced filopodia [31], we now speculate that ALIX overexpression might regulate this phenomenon by enhancing Gag ubiquitination.

**Figure 5:**
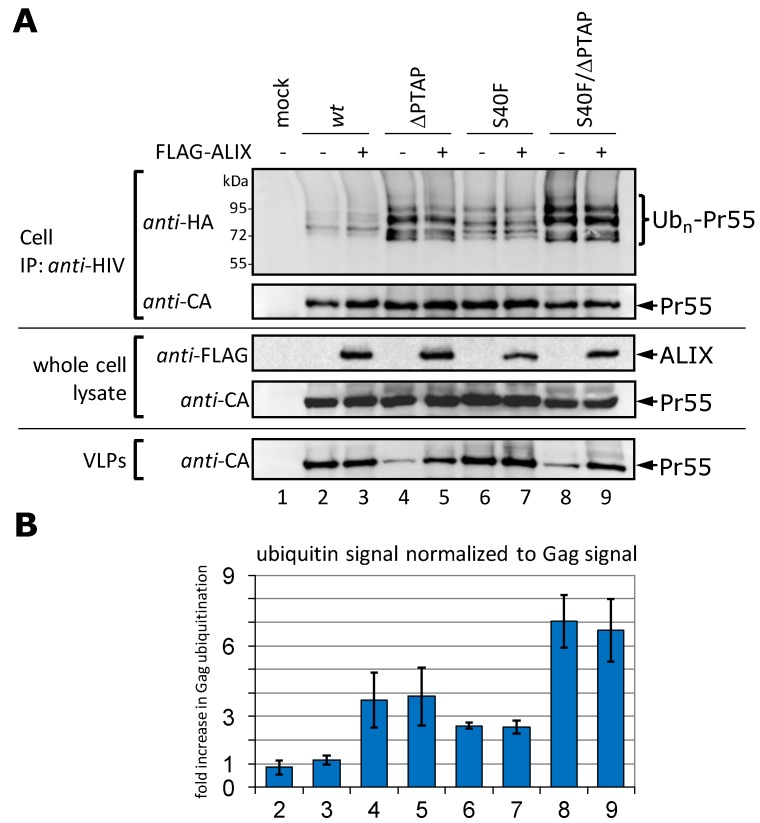
Overexpression of ALIX increases virus release but has no influence on Gag ubiquitination. (**A**) HeLa cells were co-transfected with HA-tagged ubiquitin, pΔR constructs as indicated, and either with, or without, FLAG-ALIX plasmid. Gag processing was inhibited by the addition of the PR-inhibitor Ritonavir. For detection of ubiquitinated Gag species, Gag was precipitated from whole cell lysates by immunoprecipitation using *anti-*HIV antibodies, and ubiquitin was detected by *anti-*HA staining. VLPs were pelleted from the supernatant and the amount of Gag released as VLPs was detected by *anti-*CA staining; (**B**) Evaluation of three independently performed experiments. Bars represent the arithmetic mean ± SD. The numbering is according to (A).

In order to investigate whether or not there is any correlation between the efficiency of virus release or the increased ALIX binding and Gag ubiquitination, we co-transfected HeLa cells with p∆R *wt*, the S40F and ∆PTAP mutants, as well as a S40F/∆PTAP double mutant, with or without ALIX expression plasmids. Consistent with previous findings, ALIX overexpression efficiently restored the VLP release of the ∆PTAP and the S40F/∆PTAP mutants, whereas the *wt* and the S40F mutant were not affected (Figure 5A, compare lanes 4 with 5 and 8 with 9) [12,13,30]. Analyses of Gag ubiquitination levels in cells revealed that, although ALIX overexpression could significantly enhance virus release of the PTAP-deficient constructs, it had no effect on the ubiquitination of Gag in any of the cases investigated (Figure 5A). The statistical significance of this finding was further confirmed by quantification of the ubiquitin signals from three independent experiments (Figure 5B). All together, these results indicate that the level of Gag ubiquitination does not correlate with the efficiency of virus release, now also observed in the context of ∆PTAP mutants. This notion is further supported by the observation that the S40F mutant, which exhibits *wt* virus release efficiency, becomes ubiquitinated like the release-deficient ∆PTAP mutant (Figure 5). Moreover, the respective S40F/∆PTAP double mutant shows an about two-fold increase in ubiquitination compared to the respective single mutants which might even indicate an additive effect of the S40F and ∆PTAP mutations on Gag ubiquitination (Figure 5B).

### 3.5. The S40F Mutation Increases the K48-Linked Polyubiquitination of Gag

Previous studies reported that all major processing products of Gag can be modified by attachment of ubiquitin [21,22,25,26,38]. Particularly, monoubiquitination of lysine residues in the proximity to l-domains was demonstrated to increase the affinity of Gag to TSG101* in vitro* [7]. Moreover, a recent study implicated a possible link between the S40F mutation and syntenin [31], a protein which was characterized to bind polyubiquitin irrespectively of the linkage type [33,34]. Furthermore, it was previously shown that mutation of the PTAP l-domain leads to a prolonged membrane association of Gag which subsequently induces its exposure to membrane resident ubiquitin ligases and, thus, elevates its K48-linked polyubiquitination [26,42].

Therefore, we wanted to investigate what kind of polyubiquitination is induced by the S40F mutation. We applied a system that discriminates between K48- and K63-linked polyubiquitination by using linkage-specific polyubiquitin antibodies.

**Figure 6:**
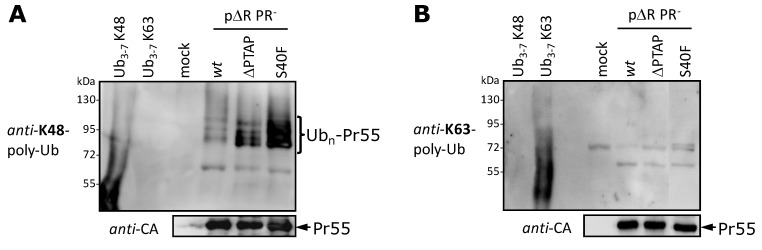
Gag is modified by K48-linked polyubiquitin. HeLa cells were transfected with p∆R PR^−^ plasmids encoding *wt* Gag, or mutants thereof as indicated. 48 h post transfection VLPs were pelleted from the cell culture supernatants through a 20% sucrose cushions and subsequently analyzed by Western blotting using antibodies specifically recognizing either K48- (**A**) or K63-linked (**B**) polyubiquitin. 100 ng of recombinant polyubiquitin chains linked either by internal lysine 48 or 63 were used as control (Ub_3-7_ K48 or Ub_3-7_ K63, respectively). Comparable amount of Gag was present in each VLP preparation as shown by staining with *anti-*CA antibodies (lower panels (A),(B)).

Due to the nature of ubiquitination at least some of the Gag-ubiquitin adducts might be inaccessible for the immunoprecipitation after de- and re-naturation of cell extracts. This disadvantage was circumvented by analyzing purified VLP preparations where all virion associated Gag-ubiquitin adducts become observable by Western blot. Analyses of *wt*, ∆PTAP or S40F VLPs using the K48-specific antibody demonstrated that even the *wt* VLPs displays a very weak baseline amount of K48-linked polyubiquitination, that was clearly enhanced when the PTAP or Ser-40 were mutated (Figure 6A). In contrast, a negative result was observed when the Western blots of the VLP fractions were stained for K63-linked polyubiquitin, although the corresponding recombinant polyubiquitin chains were specifically recognized in the same Western blot and the sensitivity was comparable to the detection of K48-linked polyubiquitin (Figure 6B).

These findings demonstrate that predominantly K48-linked polyubiquitin chains are attached to Gag and that this effect is enhanced when Ser-40 is mutated. The same phenomenon was previously described for the ∆PTAP mutant and characterized to depend on an increased membrane binding of Gag [26,42]. Furthermore, these results confirm that these K48-linked polyubiquitinated Gag species even participate in Gag assembly process for all variants, *wt*, ∆PTAP, S40F, though at different levels.

### 3.6. The S40F Mutation Enhances MHC-I Antigen Presentation of Gag

It is well established that the K48-linked polyubiquitination is a prerequisite for the entry of proteins into the MHC-I antigen presentation pathway [61]. To more precisely ensure our findings concerning the S40F mutant, we sought to use another read out system for the extent of K48-linked polyubiquitination which is the presentation of Gag derived epitopes in the context of MHC-I molecules at the cell surface. This immunochemical approach was previously proven as a highly reliable and sensitive method to follow the entry of Gag into the UPS [42,43,46,62]. As there are no antibodies available which recognize Gag-derived epitopes bound to MHC-I molecules, the ovalbumin-derived sequence SIINFEKL (SL) was introduced as a model epitope into the p2 spacer region of Gag (Gag-SL [43]). By using this method, elaborated previously for the ∆PTAP mutant [42], we wanted to investigate whether the S40F mutation, which clearly increases K48-linked polyubiquitination, also enhances the entry of Gag into the MHC-I pathway.

**Figure 7:**
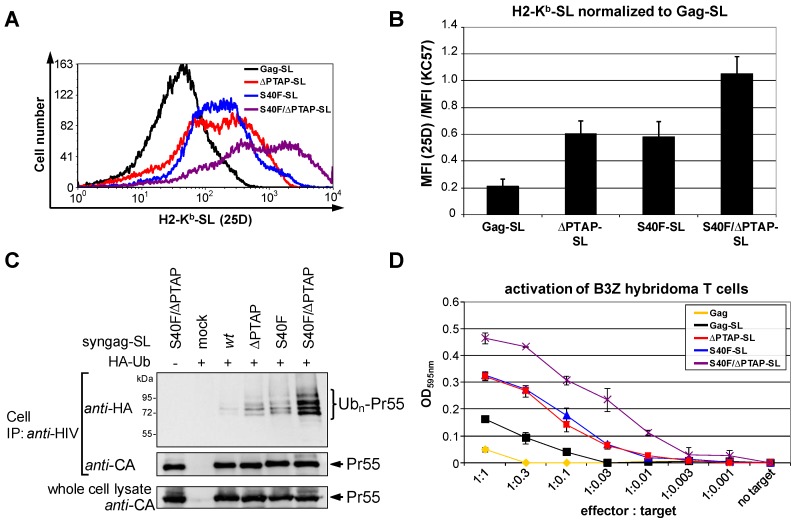
The S40F mutation induces an enhanced MHC-I antigen presentation of Gag derived epitopes. (**A**) HeLa-K^b^ cells were transfected with syngag expression constructs coding for Gag-SL, ΔPTAP-SL, S40F-SL and S40F/ΔPTAP-SL. H2-K^b^-SL complexes presented on the surface of Gag-positive cells were quantified by flow cytometry using 25D1.16-APC. A representative histogram plot is shown; (**B**) Quantification of three independent experiments. The mean fluorescence intensity (MFI) of the 25D1.16 staining was normalized to the MFI of the intracellular *anti-*Gag staining obtained after permeabilization of the plasma membrane. Bars represent mean values ± SD; (**C**) Analysis of the Gag ubiquitination of the corresponding constructs. Gag was recovered from whole cell lysates by immunoprecipitation using *anti-*HIV antibodies, and ubiquitinated species were detected by anti-HA staining; (**D**) Activation of B3Z hybridoma T cells was assessed by a colorimetric β-galactosidase assay after overnight cocultivation with HeLa-K^b^ cells expressing Gag *wt*, Gag-SL *wt*, ΔPTAP-SL, S40F-SL or S40F/ΔPTAP-SL in various effector-to-target ratios.

HeLa cells stably expressing the murine MHC-I allotype H2-K^b^ (HeLa-K^b^, [63]) were transfected with expression plasmids coding for Gag-SL, ∆PTAP-SL, S40F-SL and S40F/∆PTAP-SL. Flow cytometry using the mAb 25D1.16, which specifically recognizes H2-K^b^-bound SL, revealed that cells expressing ∆PTAP-SL or S40F-SL displayed higher numbers of H2-K^b^-SL complexes at the cell surface when compared to cells expressing *wt* Gag-SL (Figure 7A). Consistently, the S40F/∆PTAP-SL double mutant even revealed the strongest effect on SL presentation (Figure 7A). According to the rule of antigen presentation, the amount of intracellular antigen should directly correlate with the number of epitopes presented on the cell surface, provided that those antigens exhibit similar turnover rates [64]. In order to compensate for possible differences in the steady state levels of the Gag-SL variants, the mean fluorescence intensity (MFI) of the staining with 25D1.16 was normalized to the MFI of the intracellular staining for Gag. Even when the budding defect and, thus, the accumulation of cell associated Gag was taken into account, analyses of three independent experiments revealed an approximately threefold increase in SL-presentation when the ∆PTAP-SL or S40F-SL variant were compared to *wt* Gag-SL (Figure 7B). The S40F/∆PTAP-SL exhibits an even more pronounced, approximately fivefold increase in SL-presentation in comparison to *wt* Gag-SL.

It was previously well elaborated that the quantification of SL loaded K^b^ molecules on the cell surface correlates with an even more sensitive method, the activation of B3Z hybridoma T cells [46]. These indicator cells express a T-cell receptor which specifically recognizes SL bound to H2-K^b^, leading to T-cell activation and thus the induction of the *lac*Z gene expression. To investigate if the S40F mutation enhances the immunogenicity of Gag, we tested the ability of HeLa-K^b^ cells expressing Gag-SL *wt*, ∆PTAP-SL, S40F-SL or S40F/∆PTAP-SL to activate B3Z hybridoma T cells. As a negative control, HeLa-K^b^ cells were transfected with Gag *wt* without SL. After overnight cocultivation, β-galactosidase activity was measured by a colorimetric assay, showing that cells expressing ΔPTAP-SL or S40F-SL mutant were able to induce a clearly enhanced T-cell activation over a broad range of effector-to-target ratios when compared to cells expressing *wt* Gag-SL (Figure 7D). In correlation with the analysis of the Gag ubiquitination by Western blot (Figure 7C) and the MHC-I antigen presentation (Figure 7A,B), the S40F/∆PTAP-SL mutant displayed the highest potential to activate T cells* in vitro*.

Thus, these sensitive immunological methods underline that both, PTAP deletion and mutation of Ser-40 with Phe, act independently of each other, but can also exert an additive effect on the K48-linked polyubiquitination of Gag and, consequently, on its entry into the UPS.

### 3.7. Mutations of Ser-40 Do Not Change the Extent or Localization of Secondary Structure of p6

S40F, but none of the conservative exchanges, induces the phenotype described above. In order to unravel the underlying molecular rationale, we set out to investigate potential structural consequences of the mutations of Ser-40 of p6 by NMR spectroscopy. In order to achieve structural information at atomic resolution, chemical shift index (CSI) plots were created of the individual ^1^H resonances of each p6 peptide. CSI plots of the α-protons (Hα) chemical shifts relative to those of residues in a random coil have proved to be an appropriate and powerful method for determining the presence of secondary structure in peptides and proteins [50,65]. It has been shown experimentally that Hα-proton chemical shifts greater than 0.1 ppm relative to the random coil values are qualitative indicators of secondary structure [65]. A minimum of four adjacent residues with an upfield shift relative to random coil (negative CSI) is indicative of an α-helix, whereas β-sheets require a minimum of three residues with downfield shifts (positive CSI) [65].

To ascertain whether the S40F, S40N or S40D mutations alter the C-terminal helix of p6, the synthetic peptides (*s*)p6(23-52)S40F, (*s*)p6(23-52)S40D and (*s*)p6(23-52)S40N were characterised by ^1^H NMR spectroscopy. Previously, we solved the structure of the *wt* peptide *s*p6(23-52) by NMR and found that the C-terminal fragment adopts the same structure as the full length *wt* molecule *s*p6(1-52) [48]. Therefore, it was legitimate to analyze the structure of the C-terminal mutant peptides and compare it with the *wt* molecule.

**Figure 8:**
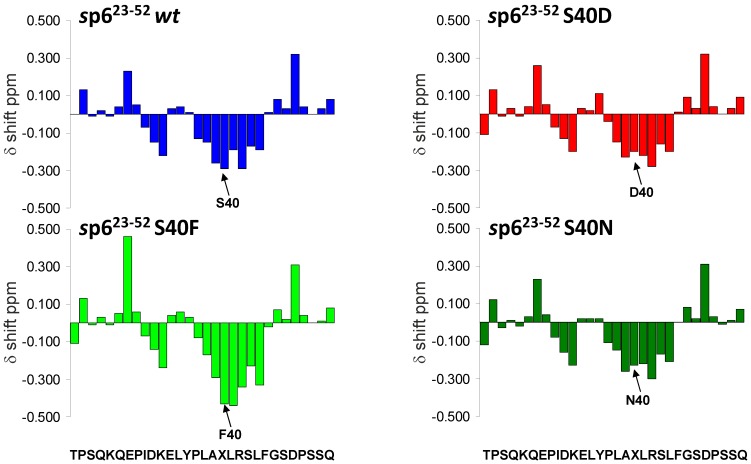
Mutations of Ser-40 do not change the extent or position of the secondary structure of the C-terminus of p6. Chemical shift differences (ppm) of the α-protons between the experimental values and those for residues in a random coil for the C-terminal peptides *s*p6(23-52) *wt* and the mutants *s*p6(23-52)S40F, *s*p6(23-52)S40D and *s*p6(23-52)S40N in 50% aqueous TFE at pH 3.

After complete assignment of the ^1^H resonances of the NMR spectra of each peptide, NOE cross peaks important for secondary structure identification, were determined. The observation of NH_i_ - NH_i+1_, NH_i_ - NH_i+2_, αH_i_ - NH_i+2_, αH_i_ - NH_i+3_, αH_i_ - NH_i+4_ and αH_i_ - βH_i+3_ NOEs, which are indicative of helical secondary structure, showed that, similarly to *wt* *s*p6(23-52), the mutant *s*p6(23-52)S40F has a preference for an α-helical structure involving residues Ile-31 to Asp-48 under hydrophobic membranous conditions (50% aqueous trifluorethanol (TFE) solution) [30]. Substitution of Ser-40 with Phe did not change the position or number of residues included in the C-terminal helix compared with *wt* *s*p6(23-52), and their structures appear to be even similar. This was confirmed by a comparison of plots of the α-proton chemical shifts relative to those of random coil values (Figure 8), which indicates that the substitution of Ser-40 with Phe slightly stabilizes the region of the C-terminal helix comprised of residues Leu-41 to Leu-44, proximal to Phe-40. The conservative mutations S40D and S40N did not provide any observable effect on the secondary structure, which like the S40F mutation, resemble that of *wt* *s*p6(23-52) (Figure 8).

### 3.8. The S40F Mutation Creates a Hydrophobic Domain Involving Aromatic Residues Tyr-36 and Phe-40 at the Same Surface of the C-Terminal Helix of p6

**Figure 9:**
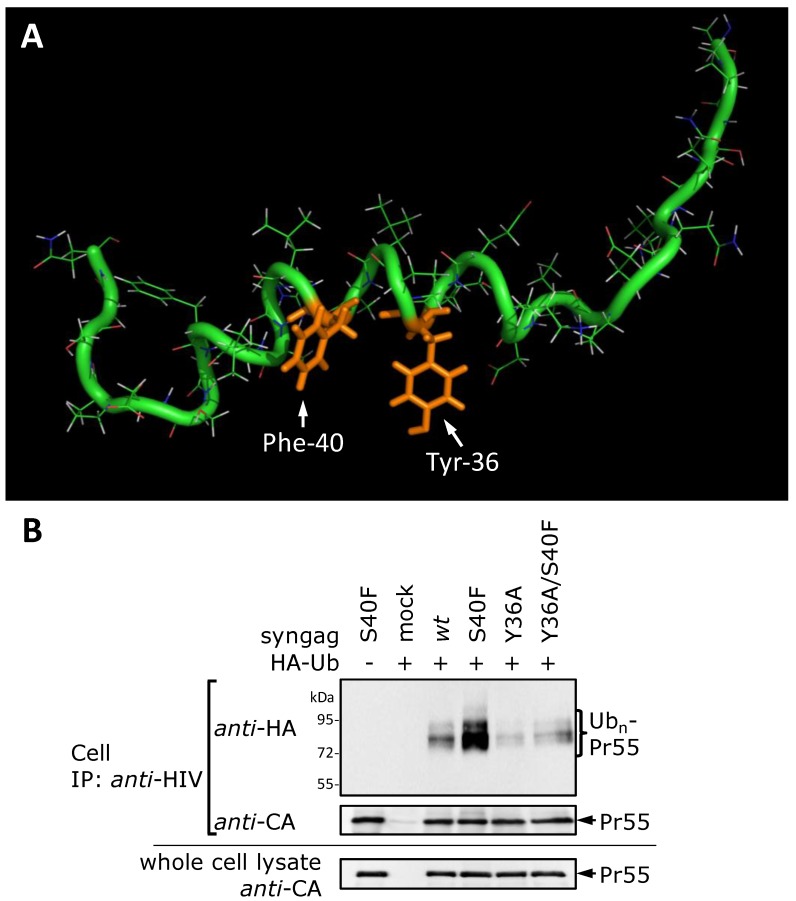
NMR structure of p6(23-52)S40F confirms formation of a new hydrophobic domain. (**A**) Structure of *s*p6(23-52)S40F determined by NMR spectroscopy. The residues Y36 and F40, which are located at the same surface of the helix, forming a new hydrophobic domain within the C-terminal helix of p6, are labeled in orange. (**B**) HeLa cells were co-transfected with HA-tagged ubiquitin and the *syngag* expression plasmids directing the expression of *wt* Gag and mutants thereof. The isolation and detection of ubiquitinated Gag species from cell fractions were carried out as described in Figure 2B. The amount of Gag recovered by immune precipitation and Gag recovered from whole cell lysate was detected with CA specific antibodies.

To rationalize the increased membrane association caused by the S40F mutation, the structure of *s*p6(23-52)S40F was calculated based on the assigned crosspeaks observed in the 2D ^1^H NOESY spectrum. The NMR structure revealed that the residue at position 40 is located at the same surface of the helix (separated by one turn of the helix), and, thus, is in spatial proximity to the bulky hydrophobic residue Tyr-36 (Figure 9A). Thus, the S40F mutation creates a hydrophobic domain involving the bulky aromatic residues Tyr-36 and Phe-40, which may account for the observed increased membrane affinity of the S40F mutant. This indicates that the C-terminal α-helix of p6 characterized by NMR spectroscopy should be also present and functionally active in HIV-1 expressing cells.

To confirm that the Tyr-36 plays a crucial role in the formation of the hydrophobic patch in Gag-expressing cells, we examined the Gag ubiquitination of the Y36A mutant, carrying an exchange in position 36 from Tyr to Ala, and the Y36A/S40F double mutant (Figure 9B). Western blot analyses revealed that the Y36A mutant alone reveals no impact on the Gag ubiquitination when compared to *wt* Gag. However, in the context of the Y36A/S40F double mutant, the Y36A mutation reverts the ubiquitination phenotype induced by the S40F mutation. Consistently with the NMR structural data (Figure 9A), Tyr-36 represents an integral component of this newly formed hydrophobic patch at the C-terminus of p6 which appears to be required for membrane interaction and ubiquitination of Gag.

## 4. Discussion

In the present study we characterize a potential membrane interacting domain, at least for the natural occurring p6 mutant S40F, which is induced by the formation of a hydrophobic patch involving Phe-40 and Tyr-36 within the C-terminal α-helix of p6. This altered membrane association subsequently induces K48-linked polyubiquitination of Gag, without affecting virus release.

It is commonly accepted that ubiquitin plays an important role in retrovirus release, though its precise function in this process remains elusive so far. High amount of free ubiquitin was found in HIV-1, simian immunodeficiency virus (SIV), murine leukemia virus (MuLV) and avian leukosis virus (ALV) particles [21,22,66]. In addition, ubiquitinated species of Gag have been reported for HIV-1, SIV, and MuLV [21,22,38]. Moreover, in the case of equine infectious anemia virus (EIAV) and prototypic foamy virus (PFV), a stable C-terminal ubiquitin fusion to Gag can function as a signal for ESCRT-recruitment in the absence of an endogenous l-domain [67,68,69].

Early acting ESCRT factors like ESCRT-I, ESCRT-II, and ALIX possess ubiquitin binding domains, and thus, ubiquitin may play an important role in their substrate recognition [20,67,70,71]. Most characterized ESCRT substrates at endosomes become monoubiquitinated [18,72,73] or K63-polyubiquitinated [74,75]. Thus, ubiquitination of Gag is believed to be beneficial to the budding process by enhancing its affinity to the ESCRT [7]. Additionally, the attachment of K63-linked polyubiquitin chains was detected in released particles derived from an artificial minimal Gag construct, and therefore is thought to be involved in HIV-1 budding, yet their presence in HIV-1 particles still remains to be demonstrated [76,77].

In case of HIV-1, it was hypothesized that assembling Gag is likely recognized by membrane resident ubiquitin ligases, and this ubiquitination of Gag in turn was proposed to cooperate with l-domains to efficiently mediate virus release by the ESCRT complex [7,26,55,78]. Based on that, it was also assumed that the prolonged membrane association, caused by impaired l-domain function, subsequently leads to an increased exposure to ubiquitin ligases and thus results in an elevated ubiquitination of Gag [26].

We now show that the membrane interaction of Gag is specifically enhanced when Ser-40 of p6 is mutated to Phe. This enhancement strictly depends on the co-existence of residues Tyr-36 and Phe-40, which are both sufficient and necessary for the formation of a hydrophobic patch within the C-terminal α-helix of p6. Furthermore, in membranous environments p6 predominantly adopts α-helical structure which is the prerequisite for the formation of this hydrophobic patch [48,58]. However, while in the context of the S40F mutation, p6 likely contributes to membrane binding, this effect may not occur in the context of *wt* Gag as in the absence of S40F the size of the hydrophobic patch might be too small to be able to contribute to membrane association. Nevertheless, an increased membrane association of Gag could at least provide one explanation for the augmented K48-linked polyubiquitination observed for the S40F mutant.

**Figure 10:**
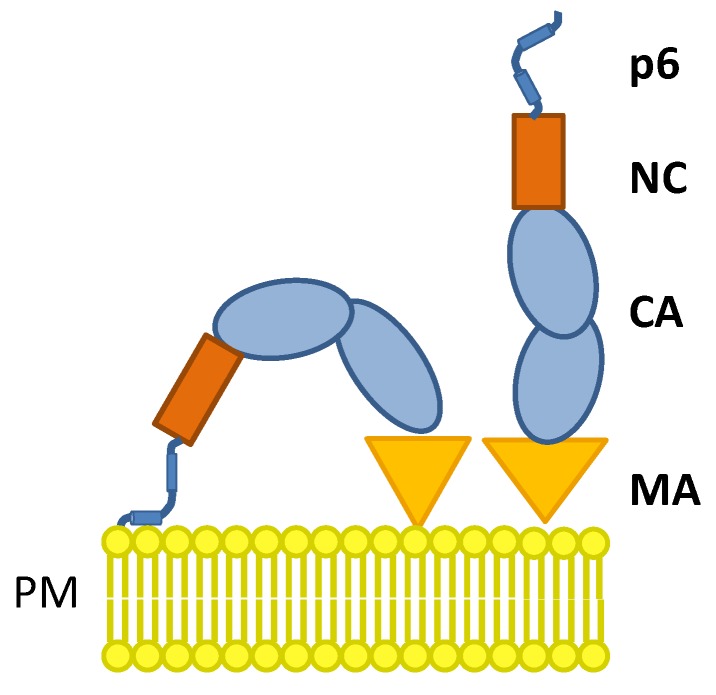
Model of the membrane interaction of Gag mediated by MA and the C-terminal α-helix of p6.

It has been previously supposed by Salgado* et al.* that the hydrophobic C-terminal α-helix of p6 inserts in plane into the lipid bilayer [36] and may, therefore, act as membrane targeting domain of Pr55, in addition to MA. This would drive at least a fraction of p6 molecules in the context of Pr55 to fold back to the membrane, a process which might even occur during assembly and consequently function as a secondary membrane binding domain of Gag (Figure 10). Most intriguingly, it has already been described that during assembly RNA associated Gag switches from a “compact” conformation to a final “stretched” conformation [79,80,81]. It would be intriguing to investigate the contribution of the S40F mutation to this process. In the compact conformation of Gag, NC is located in close proximity to MA, allowing both domains to interact with the inner leaflet of the plasma membrane by electrostatic interactions [81]. Thus, it is conceivable that in this dynamic process p6 interacts with the lipid bilayer and might also participate in the structural transition of Gag.

The membrane interaction of p6 is specifically enhanced when Ser-40 is exchanged to Phe which can be rationalized from the NMR structure of p6 showing that the residue at position 40 is in spatial proximity to the bulky hydrophobic residue Tyr-36 (Figure 9). In general, an α-helix turns 100 degrees for each residue included. That means that formally 3.6 residues are required for a full turn of the helix [82]. Subsequently, amino acids 3-4 residues apart are relatively close in space and at the same surface of the α-helix. Thus, when the polar residue Ser-40 is replaced by the bulky non-polar Phe residue, a new hydrophobic domain is formed within the C-terminal helix of p6. The residue at position 40 is relatively close in space to Tyr-36, which then creates the new hydrophobic domain as the driving force that augments membrane interaction observed selectively for the S40F mutant. When the polar residue Ser-40 is substituted with either Asn or Asp, as exemplified by the mutants S40N and S40D, it is replaced with another polar, or even a negatively charged residue at this position. Thus, the hydrophobic domain, stretching up to Tyr-36, cannot be formed by these mutants. The fact that particularly the establishment of a new hydrophobic domain at the surface of the C-terminal helix of p6 leads to increased membrane interaction is in agreement with the findings of Dathe* et al.* who reported hydrophobic domains as a determinant for membrane interaction of α-helical peptides [83].

We could neither detect an accumulation of budding virions on the cell surface nor a delay in virus release kinetics in our previous studies for the S40F mutant, which was then confirmed by Watanabe* et al.* [30,31]. Thus, it is legitimate to assume that this increase in membrane bound Gag observed for the S40F mutant occurs independently of virus release. Nevertheless, the budding competent S40F mutant, like the release defective ∆PTAP mutant, exhibits K48-linked polyubiquitination, a type of polyubiquitin chain that was never implicated in any ESCRT function. This, among other findings, indicates a role of ubiquitin and p6 distinct from promoting virus release [42]. Moreover, in the case of the S40F mutant Gag ubiquitination does not correlate with virus release and occurs independently of l-domain function. Thus, this type of ubiquitination might even originate from an ESCRT independent mechanism. This notion is further supported by the finding that overexpression of ALIX has no influence on Gag ubiquitination or virus release of the S40F mutant although it significantly enhances virus release of both the ∆PTAP mutant and the S40F/∆PTAP double mutant. In this particular case, ALIX might augment the release of Gag after its accumulation and subsequent ubiquitination at the plasma membrane.

We failed to detect K63-linked polyubiquitinated Gag in purified virions. Given that such Gag species might still exist intracellularly, those could be either hydrolyzed during the budding process by ESCRT associated ubiquitin hydrolases like AMSH, which preferentially cleaves off K63-linked polyubiquitin [84], or just be excluded from virus assembly.

Instead, we could demonstrate that virus-associated Gag is predominantly modified by K48-linked polyubiquitin, an effect which, however, was additionally enhanced when the PTAP motif or Ser-40 were mutated. The K48-linked polyubiquitination of proteins is the prerequisite for their proteasomal degradation [61]. Although the steady state amount of S40F mutant Gag was not affected, the MHC-I antigen presentation, as the final consequence of proteasomal degradation, was significantly enhanced. This finding is consistent with earlier studies where an enhanced MHC-I antigen presentation did not correlate with a diminished half life of Gag [42,43]. A possible explanation for this phenomenon might be that just a minor fraction of Gag enters the UPS without reducing the overall level of Gag that remains not ubiquitinated. In consistency with this hypothesis is the notion that only a fraction of *de novo* synthesized proteins, approximately 5% to 10% depending on conditions and cell type, are entering the defective ribosomal product (DRiP) pathway [85]. In correlation with an enhanced MHC-I antigen presentation of Gag-derived epitopes, the S40F mutant displayed an increased capacity to activate T cells* in vitro*. This result implicates that S40F mutant Gag might have the potential to elicit a more potent activation of cytotoxic T-cells* in vivo*.

One possible explanation for the proteasomal degradation of S40F mutant Gag would be misfolding induced by this non-conservative amino acid exchange. However, structural analysis of the C-terminal p6 mutant peptides by NMR revealed that these mutations do not induce any changes of the secondary structure of the protein, which in each instance is similar to that of the *wt* protein (Figure 8). Thus, we can exclude the possibility for any misfolding of p6 caused by the mutations of Ser-40 with Phe, Asn, or Asp. Hence, it is unlikely that the increased ubiquitination of HIV-Gag observed for the S40F mutant could be consequent to any potential misfolding within that domain of p6.

In addition to increasing the rate of proteasomal degradation, these K48-polyubiquitinated Gag molecules participate in the assembly of Gag and are efficiently incorporated into virus particles with still undefined consequences. This most probably indicates that the ubiquitination of Gag represents a late event in the budding process enabling a certain fraction of these Gag molecules to escape proteasomal degradation. Moreover, polyubiquitin conjugated to Gag could sterically disturb the highly ordered lattice of assembling Gag and thus exert a negative effect on distinct events during the budding process, and eventually on the auto-activation of the viral protease. Another possibility may be the direct masking of PR cleavage sites by bulky polyubiquitin adducts. This could contribute to the general processing defect observed in the context of l-domain mutants. In addition, the specific CA-SP1 processing defect of the S40F mutant [30,31] could also be caused by Gag ubiquitination as particularly the CA-SP1 junction (^359^**K**ARVL**|**AEAM) is the only cleavage site within Gag that has a lysine in close proximity. This notion is further supported by the observation that the S40F and ∆PTAP mutants display a defective Gag processing as well as enhanced levels of Gag ubiquitination, whereas the mutation of Ser-40 to Ala or Asn does neither affect Gag ubiquitination nor Gag processing [31,54].

A recent study revealed that the S40F mutation can occur naturally under anti-retroviral treatment, and even more, appears to correlate with high viral load, low CD4^+^ cell counts, and ultimate treatment failure [31]. Furthermore, the S40F mutation induces Gag-containing filopodia-like membrane protrusions which were speculated to trigger cell to cell transmission of HIV-1 under antiviral treatment. It could be hypothesized that an enhanced membrane association of Gag is the driving force behind the assembly of Gag in these filopodial structures.

Taken together, we could demonstrate that the S40F mutation leads to the formation of a hydrophobic patch which increases the membrane binding of Gag and thus triggers its polyubiquitination, most likely by membrane resident ubiquitin ligases. This K48-linked polyubiquitination occurs independently of l-domain functions and the ESCRT-mediated budding process. Moreover, polyubiquitinated Gag that is incorporated into virions might account for the defects in CA-SP1 processing, virus morphogenesis, and infectivity observed for the S40F mutant.

## Conclusions

Overall, these data support a novel model for an l-domain independent function of p6, at least observed for the S40 F mutant, which regulates the association of Gag with the plasma membrane, its K48-linked polyubiquitination, the efficiency in CA-SP1 processing, and thus the infectivity without affecting the release of progeny viruses.

## Supplementary Files

Supplementary File 1
