# Supplementary material for: Mutation of the Highly Conserved Ser-40 of the HIV-1 p6 Gag Protein to Phe Causes the Formation of a Hydrophobic Patch, Enhances Membrane Association, and Polyubiquitination of Gag
Source: Viruses. 2014 Oct 2;6(10):3738–65. doi: 10.3390/v6103738 (PMC4213559; doi:10.3390/v6103738)
Supplement: Supplementary File 1 [file viruses-06-03738-s001.docx]

Supplementary data

# Mutation of the highly conserved Ser-40 of the HIV-1 p6 Gag protein to Phe causes the formation of a hydrophobic patch, enhances membrane association, and polyubiquitination of Gag

**Friedrich Hahn^1^, Christian Setz^1^, Melanie Friedrich^1^, Pia Rauch^1^, Sara Marie Solbak^2^, Nils Åge Frøystein^2^, Petra Henklein^3^, Jörg Votteler^1,†^, Torgils Fossen^2^, and Ulrich Schubert^1,^***

**Table S1** ^1^H chemical shift of *s*p6^23-52^ S40F at 300K in 50% aqueous TFE

| Amino acid | HN | Hα | Hβ | Hγ | Hδ | Hε | NH/NH_2_ | Ar-H |
| --- | --- | --- | --- | --- | --- | --- | --- | --- |
| Thr-23 |  | 4.24 | 4.35 | 1.45 |  |  |  |  |
| Pro-24 |  | 4.57 | 2.41 | 2.13  2.03 | 3.74  3.81 |  |  |  |
| Ser-25 | 8.22 | 4.49 | 3.98  3.91 |  |  |  |  |  |
| Gln-26 | 8.32 | 4.40 | 2.19  2.08 | 2.43 |  |  | 7.45  6.69 |  |
| Lys-27 | 8.17 | 4.35 | 1.91 | 1.50 | 1.75  1.80 | 3.05 | 7.60 |  |
| Gln-28 | 8.15 | 4.42 | 2.14  2.04 | 2.40 |  |  | 7.42  6.67 |  |
| Glu-29 | 8.09 | 4.75 | 2.19  1.98 | 2.54 |  |  |  |  |
| Pro-30 |  | 4.50 | 2.33 | 2.09 | 3.78 |  |  |  |
| Ile-31 | 7.67 | 4.16 | 1.94 | 1.55  1.27  0.97 | 0.89 |  |  |  |
| Asp-32 | 8.15 | 4.62 | 2.94 |  |  |  |  |  |
| Lys-33 | 8.00 | 4.12 | 1.90  1.85 | 1.49 | 1.72 | 3.02 | 7.60 |  |
| Glu-34 | 7.90 | 4.33 | 2.17 | 2.51 |  |  |  |  |
| Leu-35 | 7.72 | 4.44 | 1.72 | 1.63 | 0.95  0.88 |  |  |  |
| Tyr-36 | 7.80 | 4.63 | 3.20  3.13 |  |  |  |  | 7.12(2/6)  6.81(3/5) |
| Pro-37 |  | 4.36 | 2.39  2.17 | 2.03  1.92 | 3.67  3.81 |  |  |  |
| Leu-38 | 7.25 | 4.21 | 1.82 | 1.72 | 1.02  0.93 |  |  |  |
| Ala-39 | 7.95 | 4.06 | 1.48 |  |  |  |  |  |
| Phe-40 | 8.28 | 4.23 | 3.16  3.03 |  |  |  |  | 7.19(2/6)  7.27(3/5) |
| Leu-41 | 8.28 | 3.94 | 1.97 | 1.62 | 0.98 |  |  |  |
| Arg-42 | 8.32 | 4.04 | 1.98  1.85 | 1.73 | 3.23  3.13 |  | 7.18 |  |
| Ser-43 | 7.87 | 4.27 | 3.95  4.02 |  |  |  |  |  |
| Leu-44 | 7.73 | 4.05 | 1.38  1.27 | 1.16 | 0.68  0.62 |  |  |  |
| Phe-45 | 8.04 | 4.64 | 3.34  3.00 |  |  |  |  | 7.31(2/6)  7.24(3/5) |
| Gly-46 | 7.98 | 4.04 |  |  |  |  |  |  |
| Ser-47 | 7.92 | 4.52 | 3.95 |  |  |  |  |  |
| Asp-48 | 8.24 | 5.07 | 3.08  2.86 |  |  |  |  |  |
| Pro-49 |  | 4.48 | 2.35 | 2.06 | 3.84  3.89 |  |  |  |
| Ser-50 | 8.00 | 4.50 | 4.00  3.93 |  |  |  |  |  |
| Ser-51 | 8.04 | 4.51 | 3.97 |  |  |  |  |  |
| Gln-52 | 8.02 | 4.45 | 2.06  2.27 | 2.40 |  |  | 7.38  7.61 |  |

**Table S2** ^1^H chemical shift of *s*p6^23-52^ S40N in 50% aqueous TFE at 300K

| Amino acid | HN | Hα | Hβ | Hγ | Hδ | Hε | NH/NH_2_ | Ar-H |
| --- | --- | --- | --- | --- | --- | --- | --- | --- |
| Thr-23 |  | 4.23 | 4.34 | 1.44 |  |  |  |  |
| Pro-24 |  | 4.56 | 2.41 | 2.13/2.03 | 3.81/3.73 |  |  |  |
| Ser-25 | 8.23 | 4.47 | 3.97/3.90 |  |  |  |  |  |
| Gln-26 | 8.32 | 4.38 | 2.18/2.07 | 2.42 |  |  | 7.45/6.69 |  |
| Lys-27 | 8.17 | 4.34 | 1.90/1.80 | 1.49 | 1.73 | 3.04 | 7.60 |  |
| Gln-28 | 8.15 | 4.40 | 2.13/2.03 | 2.39 |  |  | 7.42/6.67 |  |
| Glu-29 | 8.09 | 4.52 | 2.18/1.98 | 2.53 |  |  |  |  |
| Pro-30 |  | 4.48 | 2.32 | 2.11/2.05 | 3.82/3.77 |  |  |  |
| Ile-31 | 7.67 | 4.15 | 1.92 | 1.54/1.26/  0.95/0.92 |  |  |  |  |
| Asp-32 | 8.15 | 4.60 | 2.93 |  |  |  |  |  |
| Lys-33 | 8.00 | 4.13 | 1.91/1.87 | 1.53/1.47 | 1.73 | 3.02 | 7.60 |  |
| Glu-34 | 7.92 | 4.31 | 2.16 | 2.50 |  |  |  |  |
| Leu-35 | 7.73 | 4.40 | 1.70 | 1.60 | 0.94/0.87 |  |  |  |
| Tyr-36 | 7.80 | 4.62 | 3.17/3.14 |  |  |  |  | 2/6 7.13  3/5 6.84 |
| Pro-37 |  | 4.33 | 2.36/2.02 | 2.07 | 3.80/3.64 |  |  |  |
| Leu-38 | 7.26 | 4.23 | 1.79 | 1.71 | 0.99/0.92 |  |  |  |
| Ala-39 | 8.06 | 4.09 | 1.46 |  |  |  |  |  |
| Asn-40 | 8.09 | 4.52 | 2.72 |  |  |  | 7.21/6.32 |  |
| Leu-41 | 7.97 | 4.16 | 1.88/1.80 | 1.72 | 0.97/0.93 |  |  |  |
| Arg-42 | 8.17 | 4.08 | 1.95 | 1.81/1.73 | 3.24/3.18 |  | 7.20 |  |
| Ser-43 | 7.91 | 4.33 | 4.04/3.98 |  |  |  |  |  |
| Leu-44 | 7.80 | 4.17 | 1.61/1.57 | 1.32 | 0.85/0.77 |  |  |  |
| Phe-45 | 7.97 | 4.66 | 3.34/3.02 |  |  |  |  | 2/6 7.31  3/5 7.26  4 7.17 |
| Gly-46 | 8.02 | 4.05 |  |  |  |  |  |  |
| Ser-47 | 7.96 | 4.52 | 3.94 |  |  |  |  |  |
| Asp-48 | 8.28 | 5.07 | 3.07/2.85 |  |  |  |  |  |
| Pro-49 |  | 4.47 | 2.34 | 2.05 | 3.84 |  |  |  |
| Ser-50 | 8.01 | 4.49 | 3.99/3.92 |  |  |  |  |  |
| Ser-51 | 8.05 | 4.51 | 3.98/3.93 |  |  |  |  |  |
| Gln-52 | 8.03 | 4.44 | 2.26/2.05 | 2.39 |  |  | 7.38/6.62 |  |

**Table S3.** ^1^H chemical shift of *s*p6^23-52^ S40D in 50% aqueous TFE at 300K

| Amino acid | HN | Hα | Hβ | Hγ | Hδ | Hε | NH/NH_2_ | Ar-H |
| --- | --- | --- | --- | --- | --- | --- | --- | --- |
| Thr-23 |  | 4.24 | 4.35 | 1.44 |  |  |  |  |
| Pro-24 |  | 4.57 | 2.41 | 2.14/2.02 | 3.81/3.74 |  |  |  |
| Ser-25 | 8.23 | 4.49 | 3.98/3.91 |  |  |  |  |  |
| Gln-26 | 8.33 | 4.40 | 2.19/2.08 | 2.43 |  |  | 7.46/6.70 |  |
| Lys-27 | 8.17 | 4.35 | 1.91/1.81 | 1.50 | 1.75 | 3.04 | 7.60 |  |
| Gln-28 | 8.16 | 4.41 | 2.15/2.04 | 2.40 |  |  | 7.43/6.68 |  |
| Glu-29 | 8.10 | 4.55 | 2.18/1.98 | 2.54 |  |  |  |  |
| Pro-30 |  | 4.49 | 2.34 | 2.07 | 3.83/3.78 |  |  |  |
| Ile-31 | 7.68 | 4.16 | 1.93 | 1.55/1.26  0.96/0.92 |  |  |  |  |
| Asp-32 | 8.17 | 4.63 | 2.94 |  |  |  |  |  |
| Lys-33 | 8.00 | 4.16 | 1.92/1.87 | 1.53/1.47 | 1.74 | 3.03 | 7.60 |  |
| Glu-34 | 7.93 | 4.32 | 2.17/2.13 | 2.50 |  |  |  |  |
| Leu-35 | 7.73 | 4.40 | 1.67 | 1.57 | 0.96/0.93 |  |  |  |
| Tyr-36 | 7.73 | 4.71 | 3.13 |  |  |  |  | 2/6 7.16  3/5 6.85 |
| Pro-37 |  | 4.40 | 2.35/1.92 | 2.10/2.00 | 3.74/3.60 |  |  |  |
| Leu-38 | 7.39 | 4.23 | 1.78 | 1.71 | 1.01/0.94 |  |  |  |
| Ala-39 | 7.95 | 4.12 | 1.49 |  |  |  |  |  |
| Asp-40 | 8.21 | 4.56 | 2.89 |  |  |  |  |  |
| Leu-41 | 8.03 | 4.16 | 1.90/1.82 | 1.71 | 0.98/0.94 |  |  |  |
| Arg-42 | 8.16 | 4.10 | 1.97 | 1.83/1.74 | 3.24/3.20 |  | 7.23 |  |
| Ser-43 | 7.93 | 4.34 | 4.06/4.00 |  |  |  |  |  |
| Leu-44 | 7.83 | 4.18 | 1.62/1.58 | 1.31 | 0.85/0.78 |  |  |  |
| Phe-45 | 7.99 | 4.67 | 3.36/3.03 |  |  |  |  |  |
| Gly-46 | 8.02 | 4.06 |  |  |  |  |  |  |
| Ser-47 | 7.97 | 4.53 | 3.96 |  |  |  |  |  |
| Asp-48 | 8.29 | 5.08 | 3.08/2.86 |  |  |  |  |  |
| Pro-49 |  | 4.48 | 2.35 | 2.07 | 3.86 |  |  |  |
| Ser-50 | 8.02 | 4.50 | 4.00/3.93 |  |  |  |  |  |
| Ser-51 | 8.06 | 4.53 | 4.00/3.95 |  |  |  |  |  |
| Gln-52 | 8.04 | 4.46 | 2.27/2.06 | 2.40 |  |  | 7.39/6.63 |  |


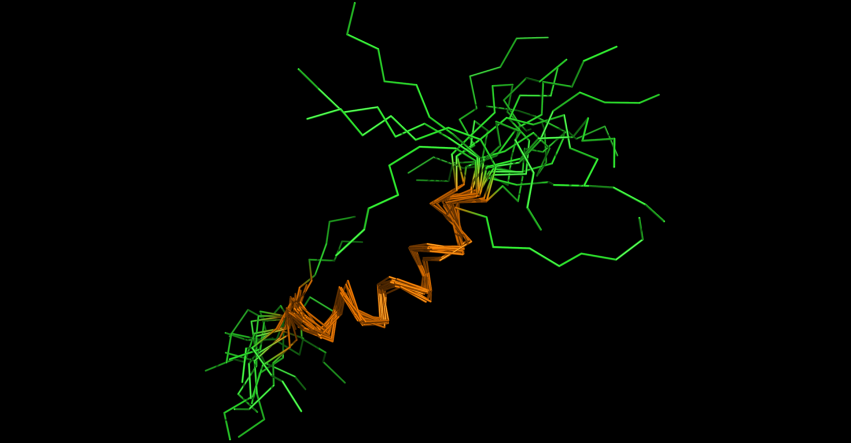


Figure S1. Superimposition of the 20 best final restrained structures of *s*p6(23-52)S40F after alignment of the backbone atoms of residues I31-D48. The residues I31-D48, which are included in the C-terminal helix of p6, are labelled in orange.
